# Oxidative Stress and Hyper-Inflammation as Major Drivers of Severe COVID-19 and Long COVID: Implications for the Benefit of High-Dose Intravenous Vitamin C

**DOI:** 10.3389/fphar.2022.899198

**Published:** 2022-04-29

**Authors:** Claudia Vollbracht, Karin Kraft

**Affiliations:** ^1^ Medical Science Department, Pascoe Pharmazeutische Präparate GmbH, Giessen, Germany; ^2^ Chair of Naturopathy, University Medicine Rostock, Rostock, Germany

**Keywords:** ascorbic acid, fatigue, oxidative stress, hyperinflammation, autoimmunity, cognitive dysfunction, Long Covid, COVID-19

## Abstract

Oxidative stress is a pivotal point in the pathophysiology of COVID-19 and presumably also in Long-COVID. Inflammation and oxidative stress are mutually reinforcing each other, thus contributing to the systemic hyperinflammatory state and coagulopathy which are cardinal pathological mechanisms of severe stages. COVID-19 patients, like other critically ill patients e.g. with pneumonia, very often show severe deficiency of the antioxidant vitamin C. So far, it has not been investigated how long this deficiency lasts or whether patients with long COVID symptoms also suffer from deficiencies. A vitamin C deficit has serious pathological consequences because vitamin C is one of the most effective antioxidants, but also co-factor of many enzymatic processes that affect the immune and nervous system, blood circulation and energy metabolism. Because of its anti-oxidative, anti-inflammatory, endothelial-restoring, and immunomodulatory effects the supportive intravenous (iv) use of supraphysiological doses has been investigated so far in 12 controlled or observational studies with altogether 1578 inpatients with COVID-19. In these studies an improved oxygenation, a decrease in inflammatory markers and a faster recovery were observed. In addition, early treatment with iv high dose vitamin C seems to reduce the risks of severe courses of the disease such as pneumonia and also mortality. Persistent inflammation, thrombosis and a dysregulated immune response (auto-immune phenomena and/or persistent viral load) seem to be major contributors to Long-COVID. Oxidative stress and inflammation are involved in the development and progression of fatigue and neuro-psychiatric symptoms in various diseases by disrupting tissue (e.g. autoantibodies), blood flow (e.g. immune thrombosis) and neurotransmitter metabolism (e.g. excitotoxicity). In oncological diseases, other viral infections and autoimmune diseases, which are often associated with fatigue, cognitive disorders, pain and depression similar to Long-COVID, iv high dose vitamin C was shown to significantly relieve these symptoms. Supportive iv vitamin C in acute COVID-19 might therefore reduce the risk of severe courses and also the development of Long-COVID.

## Introduction

Infection with SARS-CoV-2 can result in mild, moderate, severe or critical COVID-19 disease. Patients with mild illness have symptoms such as fever, cough, sore throat, malaise, headache, muscle pain, diarrhea, loss of taste and smell, but no shortness of breath; moderate illness includes evidence of lower respiratory disease with SpO2 ≥ 94%. Severe illness is indicated by SpO2 < 94%, PaO2/FiO2 < 300 mm Hg, respiratory frequency >30 breaths/min, or lung infiltrates >50%, critical illness by respiratory failure, septic shock, and/or multiple organ dysfunction (NIH, 2021).

Meanwhile, we know that three factors in particular favour severe courses: Hyperinflammation, thrombosis and immunosuppression. Excessive inflammation damages organs and the endothelium of the blood vessels. Endothelial damage and the formation of neutrophil extracellular traps (NET) favour (micro-)thromboses, which also severely impair organ function (Arcanjo et al., 2020; Ackermann et al., 2021; Blanch-Ruiz et al., 2021) and may also have impact on latent Long COVID complaints (Zhu et al., 2022).

The frequently observed lymphopenia mainly affects the T cells, which are necessary to kill virus-infected cells. The elevated ratio of neutrophils to lymphocytes predicts poor clinical outcomes in COVID-19 patients, and increased NET formation is considered a key mechanism driving airway inflammation and lung damage in this disease (Holford et al., 2020; Leppkes et al., 2020; Schönrich et al., 2020; Blanch-Ruiz et al., 2021; Dowey et al., 2021).

The increased inflammatory response has been identified as a major cause of morbidity and mortality in patients with COVID-19, and elevated concentration of pro-inflammatory markers such as cytokines IL-6, IL-18, and inflammatory chemokines IL-8, IP10 have been associated with a more severe disease outcome (Hojyo et al., 2020; Buicu et al., 2021; Satis et al., 2021; Guo et al., 2022).

## Oxidative Stress Plays a Major Role in the Immunopathogenesis in COVID-19

Oxidative stress is defined by an imbalance between increased levels of reactive oxygen species (ROS) and a low concentration/activity of antioxidants resulting in cellular damages (Preiser, 2012). ROS are both cause and consequence in the pathophysiology of the infectious process (Carr and Maggini, 2017). On the one hand ROS production is a potent mechanism for targeting infection, but on the other hand excessive ROS production can damage tissue resulting in organ damage, endothelial dysfunction, impaired lymphocyte function, and increased inflammation (Carr and Maggini, 2017; Jensen et al., 2021). Concurrently, inflammation and thrombosis cause the renewed formation of ROS, resulting in a vicious circle of oxidative stress, inflammation and disease progression. In severe COVID-19 patients, inflammatory mediators correlate with markers of oxidative stress, the strongest correlation was observed between oxidative stress index and IL-6 (Petrushevska et al., 2021).

It is particularly important to understand that oxidative stress and inflammation are mutually reinforcing each other (Jensen et al., 2021; Lage et al., 2021). This is further verified by a study on monocytes from COVID-19 patients. Persistent oxidative stress which was evident by increased mitochondrial superoxide and lipid peroxidation correlated with inflammasome activation, both cooperatively contributed to disease severity (Lage et al., 2021). The influence of oxidative stress on inflammation was further underlined by the observation that inflammasome-derived IL-1β secretion by monocytes exposed to SARS-CoV-2 *in vitro* was partially dependent on lipid peroxidation. Importantly, both increased oxidative stress and inflammasome activation persisted still after short-term patient recovery suggesting them as potential targets for host-directed therapy in order to mitigate early COVID-19 hyperinflammation and also its long-term outcomes (Lage et al., 2021). The importance of prolonged oxidative stress on long-term outcome is also evident in a murine model of sepsis, in which a long-lasting (>100 d) elevation in the basal production of ROS by immature monocytes was observed that triggered sepsis-induced immunoparalysis (Jensen et al., 2021). This could be counteracted by i. p. application of vitamin C, which prevented sepsis-associated long-lasting ROS production and reduced disease severity (Jensen et al., 2021). The long-term production of ROS was also evident in monocytes from peripheral blood samples from septic patients showing elevated ROS production both at admission and approximately 28 days later (Jensen et al., 2021). Therefore, a persistent depression of lymphocyte count and function by ROS might explain for long-term deteriorations on survival and quality of life in post-septic patients (Jensen et al., 2021). Via NF-kappaB ROS activate several processes involved in exacerbated inflammatory response and hypotension. These pathophysiological implications justified the investigation of antioxidant concomitant-therapy in animal models of sepsis and in already few clinical trials with septic patients (Prauchner, 2017).

In a recent pilot study it was observed that non-hospitalized individuals with COVID-19 are also markedly affected by systemic oxidative stress, as was reflected by reduced serum free thiol concentrations in comparison with age-, sex-, and BMI-matched healthy controls; this marker correlated with c-reactive protein (CRP) (Van Eijk et al., 2021). From another trial with 30 patients it was assumed, that the extent of neutrophil-mediated oxidative stress in plasma and albumin structural damage in serum could predict COVID-19-associated mortality (Badawy et al., 2021).

An observational study has evaluated the role of oxidative stress-related molecules in COVID-19 pathogenesis including high mobility group box-1 protein, cyclooxygenase-2 and glial fibrillary acidic protein, the receptor for advanced glycation end products. SARS-CoV-2 infection induced the upregulation of these markers in patients with the most severe forms of COVID-19. This reflects not only oxidative but also inflammatory and neurological dysfunctions in these patients (Passos et al., 2022).

The authors of a recent review describe in detail how the overwhelming production of ROS results in local or systemic tissue damage leading to severe COVID-19. Oxidative stress increases the formation of NETs and suppresses the adaptive immune system (Schönrich et al., 2020). NETosis is a particular form of cell death of neutrophils which creates extracellular networks that trap pathogens. However, these networks can also cause microembolism and play a role in the development of autoimmune reactions. The pathological effects of excessive NET-formation on immunothrombotic states are also known for sepsis, Acute Respiratory Distress Syndrome (ARDS), rheumatoid arthritis, diabetes mellitus, atherosclerosis, obesity, and cancer (Thålin et al., 2019; Stojkov et al., 2022), all these conditions go along with excessive inflammation and oxidative stress. However, the dysbalance between NET formation and degradation seems to play a central role in the pathophysiology of severe cases of COVID-19 as it is involved in inflammation, coagulopathy, organ damage, and immunothrombosis (Ackermann et al., 2021; Leppkes et al., 2020; Blanch-Ruiz et al., 2021; Al-Kuraishy et al., 2022). NETs seem to have an important impact in the amplification of the systemic inflammatory and thrombotic response because they are triggered by ROS, cytokines, and proteases and in turn induce the same *via* tissue damage etc. (Blanch-Ruiz et al., 2021).

SARS-CoV-2 starts a pathogenic cascade because it evades the IFN-I/III response (Schönrich et al., 2020). This results in prolonged and extensive replication of the virus in lung epithelial cells and in endothelial cells of the vessels. As a consequence, immune cells are massively recruited to the inflamed tissue and produce large amounts of ROS thereby creating an imbalanced oxidative stress response. This leads to damage-associated molecular patterns that trigger pro-inflammatory cytokine secretion through Toll-like receptors (TLR) signaling thereby activating the redox-sensitive transcription factor NF-κB. ROS and TLR also drive NET formation. Because of several positive feedback loops between cytokines (TNF-α, IL-1β) and ROS production as well as between cytokines and NET formation, a pathogenic cascade may result which contributes to organ damage, intravasal blood clotting, and immunosuppression (Schönrich et al., 2020). The deficits in anti-viral CD8 T cells and in CD4^+^ T cells, which are important in helping B cells to produce neutralizing antibodies and establish long-term immunity, prevent an effective decrease of the viral load. According to the authors this creates a vicious cycle that prevents a specific immune response against SARS-CoV-2 and implies a therapeutic counterbalancing of ROS by antioxidants such as vitamin C (Schönrich et al., 2020).

Excessive formation of NETs in COVID-19 is linked with the development of acute lung injury and acute respiratory distress syndrome due to the causal relationship between inflammation and NET formation through the NETs-IL-1beta loop (Al-Kuraishy et al., 2022). The signal loop may lead to prolonged inflammatory status seen in severe COVID-19 (Blanch-Ruiz et al., 2021). In a recent review, the authors investigated drugs that inhibit neutrophil recruitment or activation, prevent NETs release, specifically block NETs compounds, reduce ROS or act as an antioxidant (Blanch-Ruiz et al., 2021). Oxidative stress is one of the first inducers of NETosis as ROS induce myeloperoxidase, and antioxidants could be therefore an important supportive therapeutic option. Over 50% of the studies identified (completed or ongoing) investigate the effectiveness of antioxidants, but the authors only considered cholecalciferol, N-acetylcysteine, and vitamin D in their search (Blanch-Ruiz et al., 2021). Another review addressing the increased risk of severe COVID-19 in diabetic patients describes, among other pathogenic mechanisms, a reduced intracellular ROS production by neutrophils that weakens the immune response to infection, and an excessive extracellular ROS production, which causes damage to the host and perpetuates inflammation. The latter is mainly triggered by hyperglycemia and advanced glycation end products that induce oxidative stress and pro-inflammatory gene expression (NF-κB) in neutrophils (Dowey et al., 2021). Oxidative stress due to hyperglycemia may also be one important cause for cerebrovascular diseases in COVID-19 patients (Lou et al., 2021). In addition to the already described pro-oxidative and pro-inflammatory effects triggered by SARS-Cov-2, there is the disturbance of angiotensin-converting enzyme 2 (ACE2) due to binding to the spike protein. Elevated levels of angiotensin 2 activate NAD(P)H oxidases, which produce ROS and thus promote the activation of Nf-kappa-ß and inflammasomes (Beltran-Garcia et al., 2020). According to the authors of these reviews the plausible benefit of supportive antioxidant administration in COVID-19 should be further investigated through clinical trials.

Contrary to most ongoing clinical studies, which investigate the effects of iv vitamin C for the treatment of severe COVID-19, the start of treatment with antioxidants should be as early as possible, preferentially between days 1 and 7 of infection, to prevent ROS-induced suppression of antiviral T cells and to maintain a normal neutrophil to lymphocyte ratio. This may prevent SARS-CoV-2 infection from spreading and worsening towards Acute Respiratory Distress Syndrome (ARDS) (Schönrich et al., 2020)

## Vitamin C: Physiologic Effects, Deficiency, Bioavailability

Vitamin C encompasses the terms ascorbic acid and ascorbate, is one of the most important circulating antioxidants (Frei et al., 1989), and also a co-factor of more than 150 metabolic functions (Blaszczak et al., 2019). Ascorbate is the biologically active form that is oxidized to dehydroascorbate, when ROS are neutralized. As an enzymatic co-factor, it is particularly important for the synthesis of collagen and carnitine, the bioavailability of tetrahydrobiopterin, and thus the formation of serotonin, dopamine, and nitric oxide, the synthesis of noradrenaline, the biosynthesis of amidated peptides, the degradation of hypoxia-induced factor 1 α (HIF-1 α), and the hypomethylation of DNA (Carr and Maggini, 2017; Institute of Medicine (US) Panel on Dietary Antioxidants and Related Compounds, 2000).

The importance of vitamin C for the degradation of the transcription factor HIF-1 on the one hand and the treatment of anemia on the other hand is an interesting topic that reflects the physio-pathological complexity. Vitamin C is cofactor of HIF prolyl hydroxylase, which initiates the degradation of the transcription factor. In the absence of vitamin C, degradation is hampered and results in increased HIF-1 α concentrations (Campbell et al., 2019). Elevated HIF-1 α levels seem to play an important role in promoting SARS-CoV-2 infection by inducing pro-inflammatory responses to the virus, suppressing IFN, stimulating NET-formation due to increased glycolysis (Duan et al., 2021; Peng et al., 2021; Borella et al., 2022; Tian et al., 2021) and increasing the cardiovascular risk after SARS-Cov-2 infection (Zhang L. et al., 2021). Therefore, the increased expression of HIF 1α mRNA and its related genes in myeloid blood cells from critically ill COVID-19 patients might be an important therapeutic target (Vadillo et al., 2021), in this context one group also investigated the potential use of HIF prolyl hydroxylase inhibitors (Wing et al., 2021). Furthermore, the transcription factor HIF is important for erythropoiesis, and COVID-19 is often associated with anemia (Taneri et al., 2020), but also lymphopenia, neutrophilia, thrombocytopenia, and stress erythropoiesis (Elahi, 2022). The reasons for the dysregulated hematopoiesis in severe COVID-19 are complex and still not fully understood. An interesting point mentioned by Elahi, 2022, is a functional iron deficiency. There might be a link to vitamin C which is able to raise hemoglobin levels in patients with erythropoietin hyporesponsiveness undergoing hemodialysis due to increased iron utilization (Deved et al., 2009; Einerson et al., 2011). In this context, it is also worth mentioning that iv vitamin C significantly reduced anemia, lymphopenia, and thrombocytopenia in a retrospective oncological cohort study (Ou et al., 2020) and increased the total lymphocyte count in cancer patients with lymphopenia in an observational study (Rodríguez et al., 2017).

Certain cell types accumulate high concentrations of vitamin C *via* the sodium-dependent vitamin C transporter (SVCT). Whereas physiological plasma levels are 50–70 μM, some organs have concentrations in the millimolar range (brain 2–10 mM, adrenal glands 4–10 mM, liver and lung 1 mM) (Lykkesfeldt and Tveden-Nyborg, 2019). Leukocytes actively accumulate vitamin C against a concentration gradient *via* SVCT, resulting in concentrations >1 mM, and increase the concentration following oxidative burst up to 10 mM due to non-specific uptake of the oxidized form, dehydroascorbate *via* glucose transporters (GLUTs), which is rapidly reduced to ascorbate intracellularly (Carr and Maggini, 2017). Presumably this protects immune cells from the self-induced oxidative burst due to killing pathogens.

Any form of inflammation is associated with the formation of ROS and leads to a high consumption of antioxidants, especially vitamin C. During an acute infection vitamin C levels in leukocytes and plasma drop extensively due to increased inflammatory response and metabolic demand and require high (i.e., gram) doses of vitamin C for compensation (Hume and Weyers, 1973; Carr and Maggini, 2017). Additionally, cellular vitamin C transporters were described to be downregulated during acute inflammation (Subramanian et al., 2018).

Fatigue, weakness, pain, cognitive disorders, depression-like symptoms, bleedings, impaired wound healing, susceptibility to infections, and retarded recovery after diseases are well-known symptoms of a vitamin C deficiency (Institute of Medicine (US) Panel on Dietary Antioxidants and Related Compounds, 2000). A clinically relevant vitamin C deficiency also is a disease-eliciting condition: A major symptom of scurvy is a marked susceptibility to infections, particularly of the respiratory tract, with pneumonia being one of the most frequent complications and causes of death. The connection between pneumonia and scurvy became apparent through autopsies etc. at the beginning of the 20th century when scurvy was much more common (Hemilä, 2017).

Many critically ill patients with pneumonia, sepsis or COVID-19 suffer from clinically relevant vitamin C deficiency (Carr et al., 2017; Arvinte et al., 2020; Carr et al., 2020; Chiscano-Camón et al., 2020; Pincemail et al., 2021), which often is not corrected by the regular addition of vitamin C to enteral or parenteral nutrition (usually 200 mg/day) (Carr et al., 2017). In sepsis the deficiency of vitamin C is associated with organ dysfunction and increased mortality (Borrelli et al., 1996). So far, 6 studies suggest that vitamin C is rapidly depleted in COVID-19, and that supplemental vitamin C intake is important in the acute phase of the disease. Hypovitaminosis, i.e., vitamin C plasma levels <23 μmol/L, is found in about 70–80% of COVID-19 patients (Holford et al., 2021). The authors point out that a short-term (i.e., 2–4 days) intervention with high dose iv vitamin C may not be sufficient to achieve a persisting benefit, as 15–25% of patients may return to vitamin C deficiency again after the end of the intervention. As clinically relevant vitamin C deficiencies are difficult to be corrected through diet only, most probably this deficiency will persist during the convalescence period.

The oral bioavailability of vitamin C is limited by active, energy dependent enteral resorption and restricted further by intestinal mucosal disorders. Even with frequent high doses, the renal threshold limits the maximum plasma concentrations following oral application to 220 μM vitamin C. High vitamin C blood levels in the millimolar range therefore can only be achieved by iv application in gram amounts (Padayatty et al., 2004), allowing rapid correction of deficiencies. Studies in critically ill patients observe that a sufficiently high iv vitamin C dose (Fowler et al., 2014), administered early (Frommelt et al., 2020; Lv et al., 2020) and for a sufficiently long time (Scholz et al., 2021), are decisive for its benefit. A minimum of 2–3 g vitamin C iv per day is required to restore physiological plasma levels, but higher pharmacological dosages of 6–24 g per day may result in greater benefits, including the reduced use of vasopressors, faster recovery and lower mortality rates (Spoelstra-De Man et al., 2018; Spoelstra-De Man et al., 2019).

On the basis of the experimental and clinical studies on oxidative stress in sepsis mentioned before, the ability of iv vitamin C to reduce markers of oxidative stress in sepsis has been investigated. However, iv vitamin C did not attenuate the increase in protein carbonyls (Spencer et al., 2022), but it reduced markers of oxidative stress such as plasma lipid peroxides (malondialdehyde), and 8-hydroxy-2′-deoxyguanosine in other critical conditions such as pancreatitis, surgery and burns (Dingchao et al., 1994; Tanaka et al., 2000; Du et al., 2003; Lee et al., 2010; Pignatelli et al., 2011). However, the influence of iv vitamin C on markers of oxidative stress has not yet been investigated in COVID-19.

With respect to contraindications and warnings (such as iron storage diseases, kidney stones, renal insufficiency and glucose-6-phospate deficiency), iv vitamin C has a very good safety profile, even in critically ill patients (Spoelstra-De Man et al., 2018; Kashiouris et al., 2020; Wald et al., 2021). Due to its water solubility an excess is eliminated rapidly by the kidneys. A daily dose of 200 mg/kg body weight iv is assumed to be well tolerated (Fowler et al., 2014; Nagel et al., 2020), and is currently being tested for efficacy in randomized trials in sepsis, COVID-19 and burns [LOVIT: Lessening Organ dysfunction with VITamin C (NCT03680274), LOVIT-COVID (NCT04401150), REMAP-CAP, VICToRY: VItamin C in Thermal injury (NCT04138394)].

## Immunomodulatory Role of Vitamin C

The essential role of vitamin C for the innate and adaptive immune system is known since the 1930s: A deficiency results in impaired immunity and higher susceptibility to infections. Vitamin C has important antioxidant, immunomodulatory, and anti-infectious effects, that enable to maintain an appropriate response to pathogens without causing excessive damage to the host (Carr and Maggini, 2017; Mousavi et al., 2019). Vitamin C strengthens the body’s defense against infections *via* pleiotropic effects and protects tissues, blood vessels and the immune system against the damage by excessive inflammation. Vitamin C supports epithelial barrier function against pathogens. It accumulates in phagocytic cells, such as neutrophils, and can enhance chemotaxis, phagocytosis, generation of ROS, and thereby microbial killing. It is also needed for apoptosis and clearance of the decayed neutrophils from sites of infection by macrophages, thereby decreasing NETosis and potential tissue damage. The role of vitamin C in lymphocytes is less clear, but it enhances differentiation and proliferation of B- and T-cells, likely due to its gene regulating effects (Table 1). Reviews provide an overview of its antimicrobial, antibacterial, antiviral, antiparasitic, and antifungal properties, and highlight its importance as an antioxidant (protection of endothelium, immune cells, lung tissue etc.) and metabolic co-factor (e.g., collagen synthesis and therefore barrier function, vascular integrity), since this combination explains its immunomodulatory function (Carr and Maggini, 2017; Mousavi et al., 2019). Recently, vitamin C was shown to increase the antiviral function of lung epithelial cells *in vitro*. It significantly upregulates several metabolic pathways and interferon-stimulated genes, and downregulates pathways involved in lung injury and inflammation (Teafatiller et al., 2021).

**TABLE 1:** Immune modulating functions of vitamin C based on (Institute Of Medicine Panel On Dietary Antioxidants And Related Compounds, 2000; Carr and Maggini, 2017; Ang et al., 2018; Lee Chong et al., 2019; Mousavi et al., 2019). * important for synthesis and/or function, ↑ increased activity or synthesis on demand, ↓ reduction in pathological elevated conditions.

Immune activation	Protection against excessive inflammation
Barrier function of skin and mucosa *↑	IL-1, IL-6, TNF-α, C-reactive protein, histamine ↓
Phagocytosis, chemotaxis *↑	Oxidative stress ↓
T-, B- and NK-cell function ↑	NETosis ↓, ACE-2 (*via* IL-7↓) ↓
Interferon, immunoglobulins, complement ↑	NF-kB, hypoxia-inducible factor (HIF), DNA-, histone methylation ↓

### Supportive Vitamin C Appears to Improve Oxygenation and Reduce Inflammation in COVID-19

High dose vitamin C can alleviate symptoms in viral infections. According to Hemila, daily oral doses of 6–8 g are significantly more effective than 3–4 g (Hemilä, 2017). In addition, vitamin C supplementation reduces the risk of severe respiratory diseases such as pneumonia (Hemila and Louhiala, 2013). A recent meta-analysis demonstrated that vitamin C as an add-on to standard therapy (ST) alleviates symptoms and significantly accelerates healing of viral infections (Ran et al., 2020).

In their recent review, Holford et al. analysed the level of evidence supporting substitution of vitamin C on COVID-19. They identified 3 studies on oral application (2 randomised controlled trials (RCT), 1 retrospective cohort study), and 9 on iv application (3 RCT and 6 retrospective cohort studies) (Holford et al., 2021).

In a systematic literature research on the same subject we identified three further studies on iv vitamin C, two RCT and one retrospective study (Tehrani et al., 2021; Zheng et al., 2021; Yang et al., 2022). By now iv vitamin C as add-on to ST has been evaluated in altogether 1,578 in-patients with moderate to severe stages of COVID-19 in 12 clinical studies (5 RCTs, 7 retrospective controlled cohort studies). Table 2 shows the completed studies, differentiated according to RCT and retrospective cohort studies, in which intravenous vitamin C (IVC) was used in addition to ST. Special attention was paid here to the vitamin C dose and application duration (column 2) and the tolerability of IVC (column 4), whereby the exclusion parameters (EP) were also given for the latter.

**TABLE 2:** Clinical studies on patients with moderate to severe stages of COVID-19 with add-on IVC to standard therapy (ST).

Patient population: Number of patients and severity of COVID; mean age ±SD (years); reference	IVC intervention: dosage; duration	Comparison of outcomes: ST + IVC group vs. ST group	Tolerability of IVC: Vitamin C related exclusion parameters (EP) adverse events (AE)
Randomised controlled trials			
56 patients with COVID-19-pneumonia and multiple organ injury; 67 ± 13; (Zhang et al., 2021)	IVC 2 x 12 g/day (*n* = 27) + ST or ST + placebo (*n* = 29); 7 days	No differences in invasive ventilation-free days in 28 days (median): 26.5 vs. 10.5 days, *p* = 0.567 (primary outcome):	EP: Allergy to vitamin C, pregnancy, breastfeeding, G6PD-defiency, end-stage pulmonary disease
		Improved oxygenation at day 7: PaO_2_/FiO_2_: 229 vs. 151 mmHg, *p* = 0.01; lower IL-6: 19.42 vs. 158.00 pg/ml, *p* = 0.04	AE: non reported; serum creatinine between day 1 and 7: Slight decrease in IVC group: 64.20(46.58–85.45) to 57.50(39.95–71) µmol/L, stable in placebo group: 64.20(52.00–81.70) vs. 63.50(51.70–104.50) µmol/L
		Lower ICU mortality (%) in patients with baseline SOFA score ≥3: 27,7 vs. 52,4, *p* = 0,04	
150 patients with severe COVID-19; 52 ± 11/53 ± 12; (Kumari et al., 2020)	IVC 50 mg/kg/day + ST or ST (*n* = 75/group); no information available	Earlier symptom-free (days) (mean ± SD): 7.1 ± 1.8 vs. 9.6 ± 2.1, *p* < 0.0001	EP, AE: No information available
		Fewer days spent in hospital (mean ± SD): 8.1 ± 1.8 vs. 10.7 ± 2.2, *p* < 0.0001)	
		Need for mechanical ventilation/mortality: No difference	
60 patients with severe COVID-19 57.53 ± 18.27/61 ± 15.90; (JamaliMoghadamSiahkali et al., 2021)	IVC 4 x 1.5 g/day + ST or ST (*n* = 30/group); 5 days	Improved SpO_2_ (%) on day 3 (median): 90.5 vs. 88.0, *p* = 0.014	EP: Pregnancy, G6PD-deficiency, end-stage renal disease
		More days of hospitalization (median): 8.5 vs. 6.5, *p* = 0.028	AE: None reported
		Length of ICU stay/mortality: No difference	
54 patients with moderate to severe COVID-19 (44 analysed); 58 ± 19/61 ± 17; (Tehrani et al., 2021)	IVC 4 x 2 g/day + ST (*n* = 18) or ST (*n* = 26); 5 days	Improved SpO_2_ (%) on day 6 (mean): 90 ± 3 vs. 87 ± 5, *p* = 0.021	EP: Allergy to vitamin C, pregnancy, breastfeeding, shortness of breath due to cardiogenic pulmonary edema, chronic renal failure, diabetic ketoacidosis, history of nephrolithiasis
		Decreased respiratory rate on day 6 (mean): 24 ± 3 vs. 28 ± 4, *p* = 0.028	AE: No information available
		Length of hospital stay/mortality: No difference	
		Lower post treatment fibrosis in CT: 7% vs. 35%, *p* = 0.023	
		Pulmonary CT stage: No difference	
60 patients with moderate or severe COVID-19; 46.1 ± 9.2 to 50.3 ± 9.5; Yang et al., 2022	IVC 2 x 10g/60 kg/d + ST and TCM; no information available on duration	Faster disease recovery in severe patients: 15.89 ± 4.06 versus 13.45 ± 3.11 d, *p* < 0.05; faster symptom disappearance: moderate disease: 5.70 ± 1.16 versus 4.10 ± 0.88 d, severe disease: 13.40 ± 2.76 versus 10.20 ± 1.75 d, *p* < 0.05; faster viral clearance in moderate disease: 6.30 ± 0.95 versus 5.10 ± 1.06 d, p < 0.05; faster chest CT improvement in severe patients: 14.50 ± 2.45 versus 11.00 ± 3.06 d, *p* < 0.05	EP: Allergy to vitamin C, pregnancy, breastfeeding.AE: None reported
Retrospective cohort studies			
76 patients with moderate to severe COVID-19; median 61 (52–71); (Gao et al., 2021)	IVC 2 × 6 g on first day, 6 g/day for the following 4days + ST (*n* = 30) or ST (*n* = 46); 5 days	Improved oxygen support status: 64 % vs. 36% lower risk of 28-day mortality: HR = 0.14, 95% CI, 0.03–0.72, *p* = 0.037; lower CRP, PCT, and IL-8 concentrations	EP: Pregnancy, breastfeeding; AE: thrombocytopenia, increased total bilirubin, respiratory failure or ARDS were more common in ST than in IVC: No vitamin C-related AEs
110 patients with moderate to severe COVID-19-pneumonia; median 36 (31–47)/36 (31–46), *p* = 0.96; (Zhao et al., 2021)	IVC 100 mg/kg/day + ST or ST (55 per group); 7 days	Fewer patients progressing to severe type: 4 vs. 12; RR 0.28 [0.08, 0.93], *p* = 0.03;reduction in duration (*p* < 0.001) and incidence of SIRS (*p* = 0.008); lower CRP concentration (*p* = 0.005); lower activated partial thromboplastin time (*p* = 0.02); higher CD4 + (helper) T cells (*p* = 0.0004)	EP: Pregnancy; AE: No information available
232 patients with COVID-19-pneumonia; 60 ± 14; (Suna et al., 2021)	IVC 2 g/day + ST (*n* = 153) or ST (*n* = 170); 3 days	Shorter duration of hospital stay: 7 vs. 8 days, *p* = 0.05; no difference in re-admission rate, admission to ICU, need for advanced oxygen support, mortality; higher need for advanced medical treatment in the IVC group (*p* < 0.001)	AE: None reported
397 severely ill patients with COVID-19; 67 (61–74); (Zheng et al., 2021)	IVC 2–4 g/day + ST (*n* = 70) or ST only (*n* = 327); median 8.8 days	No significant benefit on mortality rate (primary outcome) and clinical improvement	AE: None reported
34 severely ill patients with COVID-19; 65 ± 12; (Li et al., 2021)	IVC 4 x 1.5 g/day (*n* = 8) or ST only (*n* = 24); 4 days	Higher rates of hospital mortality (19 [79%] vs. 7 [88%], *p* = 0.049) and SOFA scores (12.4 + 2.8 vs. 8.1 + 3.5, *p* < 0.005); no difference in daily vasopressor requirements or length of stay in the ICU	EP: Pregnancy; AE: No information available
236 patients with severe COVID-19; 66 (57–73); (Xia et al., 2021a)	IVC 4 x 100 mg/kg on day 1; 2 x 100 mg/kg for the next 5 days (n = 85) + ST or ST (n = 151); 6 days	Lower inflammatory markers (CRP, *p* = 0.032; IL-6, *p* = 0.005; TNF-α, *p* = 0.015)	EP: Pregnancy; AE: No information available
113 patients with severe COVID-19 and cardiac damage; 68 (59–77); (Xia et al., 2021b)	IVC 4 x 100 mg/kg on day 1, then 2 x 100 mg/kg for the next 5 days (*n* = 51) + ST or ST (*n* = 62); 6 days	Improvement in cardiac injury (OR 2.42 [1.02, 5.73], *p* = 0.04); lower levels of inflammatory markers at 21 days of hospitalization (hs-CRP, *p* = 0.036; IL-6, *p* = 0.029; IL-8, *p* = 0.047; TNF-α, *p* = 0.037)	AE: No information available

IVC, intravenous vitamin C; PaO_2_/FiO_2_: ratio of arterial partial pressure of oxygen to fraction of inspired oxygen, ICU, intensive care unit, SOFA, sequential assessment of organ failure; HR, hazard ratio; CRP, C-reactive protein; CT, computed tomography; PCT, procalcitonin; IL, interleukin; RR, relative risk; SIRS, systemic inflammatory response syndrome; TCM, Traditional Chinese Medicine; TNF, tumor necrosis factor; G6PD-deficiency, glucose-6-phosphate dehydrogenase deficiency.

The current state of evidence from these studies suggests that add-on iv vitamin C compared to ST alone seems to improve oxygenation and to reduce inflammatory markers. Depending on the respective study, this is expressed by less post treatment pulmonary fibrosis (Tehrani et al., 2021), in a faster recovery (Kumari et al., 2020), fewer days in hospital (Kumari et al., 2020), a lower risk of severe courses (Zhao et al., 2021b) or a lower mortality (Gao et al., 2021; Zhang et al., 2021).

Only in one small retrospective study increased mortality was observed in the group receiving 1.5 g vitamin C iv every 6 h additionally to ST (Li et al., 2021). However, this study has major flaws such as unequal group distribution (8 patients in the vitamin C group; 24 in the ST only group), unequal baseline conditions [mean sepsis-related organ failure assessment (SOFA) score at the beginning of therapy was almost 3 points higher in the vitamin C than in the control group (9.4 ± 3.2 vs. 6.6 ± 3.5; *p* = 0.06)]. Also, more patients in the ST only group received convalescent plasma and prednisolone.

The presently available data on vitamin C in SARS-CoV-2 infections seem to display dose relevant aspects. Studies that observed a benefit from iv vitamin C used ≥6 g daily, whereas in a study with a lower dose no effects were reported.Whereas studies with a daily dose of ≥6 g Vitamin C observed reduced pulmonary involvement and improved clinical symptoms, the retrospective study with doses of 2–4 g vitamin C per day did not reveal a significant benefit (Zheng et al., 2021).

Despite of the rather low quality of these studies due to small patients numbers, varying standard therapies and partially incomplete reporting, the results hint to a presumably dose-dependent protective effect of iv vitamin C with respect to pulmonary function and possibly even to pulmonary structure.

### Vitamin C May Reduce the Risk of Severe COVID-19

Viral infections involve a viral replication phase, followed by an inflammatory phase that can evolve into hyperinflammation leading to immune dysfunction. Vitamin C is useful in both phases as it activates many anti-viral processes and has anti-inflammatory effects mainly due to its anti-oxidative potential. This supports an early therapeutic use of antioxidants such as vitamin C also in COVID-19 in order to reduce the risk of severe courses (Holford et al., 2020; Schönrich et al., 2020; Holford et al., 2021). In a retrospective before-and-after case study supportive vitamin C infusions of 100 mg/kg body weight (bw) (i.e., 7.5 g for a 75 kg person) for 7 days were integrated into the standard protocol in mid-March 2020. Vitamin C was an add-on to other procedures such as antivirals, nutritional support, low molecular weight heparin, antibiotics, glucocorticoids, etc., which were prescribed as needed. The relative risk of developing a severe course was reduced by 72% in the vitamin C group (*p* = 0.03); as were the duration and frequency of systemic inflammatory syndrome (SIRS) and CRP levels. In addition, the blood clotting time improved, and the number of CD4^+^ (helper) T cells recovered more quickly (Zhao et al., 2021b).

Meanwhile, the first meta-analysis on iv vitamin C in COVID-19 was published, which includes 7 controlled trials (3 RCT and 4 retrospective cohort studies) (Ao et al., 2022). In this analysis, iv vitamin C missed the significance level regarding reduction of disease severity (odds ratio (OR), 0.70; 95% CI, 0.45 to 1.07; *p* = 0.10) or mortality (OR, 0.64; 95% CI, 0.41 to 1.00; *p* = 0.05) compared with placebo treatment or usual care (Ao et al., 2022). However, although the significance level was marginally missed, a clear tendency towards a reduction in mortality in the vitamin C groups is visible. The underlying studies of the analysis partially differed strongly with regard to the vitamin C dosage used [range between 24 g/d for 7 days (Zhang et al., 2021) and 2 g/d for 3 days (Suna et al., 2021)]. When calculating the effects on mortality, the (retrospective) study with the lowest dose and therapy duration (Suna et al., 2021) was weighted at 41.9% due to high patient numbers, which substantially could have influenced the outcome (Ao et al., 2022).

The importance of the dosing specially in critically ill patients is highlighted by a recent meta-analysis on iv vitamin C in sepsis that identified 15 studies involving 2,490 patients (Patel et al., 2021). Without considering the dose, only a slight superiority in terms of reduced mortality was shown for vitamin C (relative risk (RR), 0.87; 95% CI, 0.75–1.00; *p* = 0.06; test for heterogeneity I2 = 6%). When differentiating according to the dose applied, a significant reduction in overall mortality was seen for high-dose iv vitamin C (RR, 0.70; 95% CI, 0.52–0.96; *p* = 0.03), whereas low-dose iv vitamin C had no effect (RR, 0.94; 95% CI, 0.79–1.07; *p* = 0.46; test for subgroup differences, *p* = 0.14) (Patel et al., 2021).

### Recommendations Regarding Dosage of iv Vitamin C

In the clinical studies in hospitalized patients conducted so far, various iv vitamin C doses were used (table 2): Either absolute daily doses between 2 and 24 g subdivided in multiple doses, or bw-adjusted doses of 50 up to 400 mg/kg were applied.

Most frequently, absolute doses of 6–8 g or adjusted doses of 100 mg/kg bw were used. The latter corresponds to 7.5 g per day for a 75 kg person.

Authors from the Shanghai Public Health Clinical Center recommend routine administration of vitamin C on admission depending on the severity of the disease for 7 days: 100 mg/kg bw in moderate, 200 mg/kg bw in severe and 300 mg/kg bw in critical conditions. In case of aggravation, they recommend a dose increase as rescue therapy (in moderate type: 400 mg/kg for 1 day, followed by 200 mg/kg per day for another 7 days; in serve type: 600 mg/kg for 1 day, followed by 300 mg/kg per day for another 7 days; in critical type: the dosage of 300 mg/kg bw is continued until the disease improves) (Zhao et al., 2021a).

In the available studies on iv vitamin C in COVID-19, no adverse drug reactions related to vitamin C were reported (table 2). The good tolerability of high doses of vitamin C, considering the known contraindications and warnings such as pregnancy, breast feeding, kidney stones (oxalate), renal insufficiency, G6PD deficiency, is confirmed by other studies and surveys (Padayatty et al., 2010; Carr and Cook, 2018; Spoelstra-De Man et al., 2018). Many of the contraindications known for iv vitamin C were among the exclusion criteria in the RCT conducted for COVID-19 (table 2).

## Definition and Pathogenesis of Post/Long Covid Conditions

According to the WHO, post COVID-19 condition occurs in approx. 10% of individuals with a history of probable or confirmed SARS CoV-2 infection, usually 3 months from the onset of COVID-19 with symptoms, which last for at least 2 months and cannot be explained by an alternative diagnosis. The symptoms can be versatile and overlapping and include most commonly fatigue, shortness of breath, and cognitive dysfunction. They may be of new onset following initial recovery from an acute COVID-19 episode or persist from the initial illness. Symptoms may also fluctuate or relapse over time. The development and severity of Long COVID symptoms do not seem to correlate with the extent and nature of symptoms during the acute phase of the infection, although they are more common in hospitalized patients (World Health Organization, 2021a; World Health Organization, 2021b; Rajan et al., 2021).

A recent state of the art review considers excessive inflammation and oxidative stress to be main factors leading to fibrosis, especially in the lungs and heart, thrombosis, autonomic nervous system dysfunction and autoimmunity that affect multiple systems. The consequences, such as tissue injury and hypoxia, in turn increase inflammation and oxidative stress (Crook et al., 2021).

Since the ACE2 receptor is expressed on neurons, viral infection by SARS-CoV-2 could have direct negative effects on the autonomic nervous system. A complex combination of infection, a proinflammatory response induced by the autonomic nervous system and a degree of autoimmunity may contribute to the development of autonomic dysfunction (Crook et al., 2021). Autonomic dysfunction can affect particularly the cardiovascular, gastrointestinal, urogenital and thermoregulatory systems.

The immune dysregulation can lead to viral persistence and long-term infection (World Health Organization, 2021a; World Health Organization, 2021b; Rajan et al., 2021). Hyperinflammation is a central trigger point that causes endothelial dysfunction and autoimmune reactions. In order to kill the virus, immune cells produce cytokines and ROS which are also harmful for the patient, especially if not counter-balanced by the production of anti-inflammatory cytokines and antioxidants. The consequences of infection with SARS-CoV-2 are presumably especially widespread, particularly the cardiovascular complications because of the widespread presence of angiotensin-converting enzyme 2 (ACE-2) receptors, which are highly expressed in the heart and lungs and act as the binding site for coronaviruses, including SARS-CoV and SARS-CoV-2 (Crook et al., 2021; Rajan et al., 2021). Experimental transcriptome analysis reveals that ageing and chronic inflammation increase the expression of ACE-2 in lung, heart and aorta due to NF-kB and subsequent IL-7 elevation. The ACE-2 increase was blocked by vitamin C (Ma et al., 2020). Furthermore, NETs can have also great impact on Long COVID related disorders, such as aberrant immunity, neurological disorders, and lung fibrosis (Zhu et al., 2022).

The predictors of increased long COVID risk are currently being investigated. A prospective multicenter cohort study (*n* = 215) observed an immunoglobulin (Ig) signature, with low IgM and IgG3 levels, in combination with age, bronchial asthma, and number of symptoms during primary infection as predictors that increase the risk for Long COVID (Cervia et al., 2022). A multi-omic, longitudinal investigation of 309 COVID-19 patients resolved type 2 diabetes, SARS-CoV-2 RNAemia, Epstein-Barr virus viremia, and specific auto-antibodies as anticipating risk factors (Su et al., 2022). Mentionable subclinical auto-antibodies negatively correlated in this study with anti-SARS-CoV-2 antibodies. An observational study that included data from seven prospective cohort studies from six Northern European countries with almost 250,000 study participants, of whom almost 10,000 developed COVID-19 but did not require hospitalization describes especially anxiety and inflammatory processes during the acute infection as possible reasons for mental health morbidity. Severe acute COVID-19 illness-indicated by extended time bedridden is associated with increased risk for depression and anxiety symptoms whereas individuals diagnosed with COVID-19 but never bedridden due to their illness were consistently at lower risk for depression and anxiety (Magnusdottir et al., 2022). Collectively these data suggest the involvement of psychosocial factors, immune dysregulation, and chronic inflammatory conditions such as asthma and diabetes in the development of long COVID.

## The Role of Oxidative Stress in Long COVID

Oxidative stress is presumably strongly implicated in the pathophysiology of all factors causing Long COVID and its symptoms. ROS trigger inflammation, damage the endothelium, lead to microthrombi and neuroinflammation, promote the formation of autoantibodies and disrupt neurotransmitter assembly. Oxidative stress could be, in a sense, an important cause of Long COVID and thus a driving force in an immuno-endothelial-neurological vicious circle (Vollbracht and Kraft, 2021a). Nerve and immune cells contain the highest concentrations of vitamin C of all cells in order to protect themselves e.g. from oxidative stress (Carr and Maggini, 2017; Lykkesfeldt and Tveden-Nyborg, 2019). Reactions between these two cell types are bidirectional, a malfunction is easily transmitted from each of the systems to the other.

The main biological systems involved in the stress response, the hypothalamic–pituitary–adrenal axis or the autonomic nervous system, are also important in the regulation of the immune response (Mondelli and Pariante, 2021). Therefore, it is rational to consider combining strategies that reduce the levels of stress, including psychosocial intervention, and to support a modulatory immune response in order to prevent viral persistence. In a recent publication, the American psychiatrist Cohen claimed better integration of the knowledge from other upper respiratory tract infectious diseases into the treatment of COVID-19. Factors that interfere with infection defence are smoking, low vitamin C intake, and chronic psychological stress whereas it is supported by social integration, social support, physical activity, and restful sleep (Cohen, 2021). The causal involvement of stress and trauma in chronic fatigue syndrome (CFS) is well understood. Also CFS, which is associated with very similar symptoms as Long-COVID, is often triggered by an infection occurring during a time of increased mental or physical stress (Bleijenberg and van der Meer, 2018). Authors of a study on Long-COVID in England describe a strong similarity to post-traumatic stress disorder (PTSD) [27]. Both CFS and PTSD are associated with oxidative stress which causes neuronal damage in the hippocampus, amygdala and frontal cortex, being responsible for regulation of stress, emotions, anxiety and memory processing (Lee et al., 2018; Morris et al., 2018; Kim et al., 2020). Vitamin C administered in animal models is able to reduce the increase in oxidative stress in the hippocampus induced by post-traumatic stress and thereby to attenuate memory impairment (Alzoubi et al., 2020). Neurotoxicity is also mediated by intra-vascular inflammation and (micro)-thromboses that activate the microglia in the brain and trigger a persistent neuroinflammation. As a result, the finely balanced neurotransmitter assembly is severely disturbed. There is a lack of neurotransmitters such as serotonin, dopamine and noradrenalin, while the concentrations of kynurenine, quinolinic acid and glutamate rise rapidly. This finally triggers a stimulus overload and the apoptosis of neurons (excitotoxicity). Neuropsychiatric symptoms such as attention and cognitive deficits, new-onset anxiety and depression differ depending on the Brodmann area involved (Boldrini et al., 2021). High-Dose vitamin C may prevent secondary brain damage after stroke via epigenetic reprogramming of neuroprotective genes (Morris-Blanco et al., 2022)

Endothelial dysfunction induced by inflammation and oxidative stress triggers autoimmunity. There is a tendency of COVID-19 patients to develop more than 15 separate types of autoantibodies and 10 distinct autoimmune diseases such as antiphospholipid syndrome (APS), Guillain-Barre syndrome, or Kawasaki disease. Anosmia could be also a manifestation of autoimmunity (Dotan et al., 2021). NETosis is one important part that contributes to the formation of autoantibodies; another one is the molecular resemblance between self-components of the host and the virus (Dotan et al., 2021). Of particular interest is the APS associated with thrombosis, which shows strong parallels to COVID-19 induced thrombosis (Tung et al., 2021). Anti-phospholipids are not thrombogenic per se, thrombogenesis additionally requires endothelial dysfunction. SARS-CoV-2 causes oxidative stress, which damages the endothelium, and alters protein epitopes, making them more immunogenic (Tung et al., 2021). However, epitopes with a high risk of autoantibody formation play a role not only in SARS-CoV-2 itself, but also in vaccines (Dotan et al., 2021; Karami Fath et al., 2021; Chen et al., 2022).

Until now oxidative stress and vitamin C deficiency have not been investigated in patients with Long COVID. However, since both items are already well investigated and confirmed in acute COVID-19 (Holford et al., 2021), and since compensating for the vitamin C deficiency by diet alone is difficult, persistent vitamin C deficiency is likely in Long COVID. This should be investigated.

## Plausibility and Feasibility of iv Vitamin C for Fatigue

Autoimmunity, chronic viral infections, and cancer are recurrently associated with fatigue. All these conditions are highly associated with oxidative stress, inflammation, disorders of blood flow and neurotransmitter metabolism all of them contributing to fatigue.

In a recent systematic review, we evaluated the feasibility of high-dose iv vitamin C in the treatment of fatigue and identified 9 clinical trials with 720 participants (Vollbracht and Kraft, 2021b). The underlying disease that caused fatigue was cancer in most of the studies, with one study each investigating the effects also in herpes zoster, allergies, post-operative condition and in supposedly healthy full-time workers. Three of the 4 controlled trials observed a significant decrease in fatigue in the vitamin C group compared to the control group (*p* < 0.005). A decline in fatigue was also observed in the observational and pre/post studies, and the treatment effect was significant in the 4 studies with statistical comparison of pre-post values. Associated symptoms of fatigue such as sleep disturbance, cognitive impairment, depression, and pain were also commonly relieved in patients receiving iv vitamin C. Its benefit can be explained by the rapid correction of deficiency states, the effect as a co-factor of enzymatic reactions (e.g., neurotransmitter synthesis), and anti-oxidative and anti-inflammatory effects (Carr et al., 2014). For these effects the extremely high doses of vitamin C (>50 g/per infusion) are not required which are often used in cancer treatment for maximisation of its chemotherapeutic potential. In the observational study on herpes zoster (Schencking et al., 2012), a vitamin C dose of 7.5 g was applied iv 2–3 times a week. In addition to pain reduction, a significant improvement in tiredness and cognitive dysfunction was observed. A similar dose was used in a study on breast cancer patients (Vollbracht et al., 2011) and in the treatment of allergies (Vollbracht et al., 2018), where fatigue is also a symptom—affecting the quality of life. While the change in fatigue was only evaluated after 3 or more weeks in most studies, the study in apparently healthy full-time workers reported an acute reduction in fatigue (Suh et al., 2012), and one of the cancer studies observed a significant relief after 1 week treatment (Yeom et al., 2007). The use of iv vitamin C in Long COVID-associated fatigue has not yet been studied.

## Conclusion and Outlook

As oxidative stress plays a pivotal role in the pathophysiology of COVID-19 as well as in Long COVID, the therapeutic use of antioxidants in affected patients should be further addressed by RCTs. This is underlined by 6 small studies demonstrating hypovitaminosis C in plasma or serum during acute COVID-19 (Holford et al., 2021).

A possible benefit of high dose iv vitamin C has already been shown in 11 clinical trials on patients with moderate to severe COVID-19. However, the duration of application was only 4–8.8 days, and circulating vitamin C concentrations have not been investigated in these studies. A recurrent deficit after the end of treatment therefore is likely. In Long COVID, there are still no studies available on markers of oxidative stress and antioxidants. We therefore suggest monitoring of circulating vitamin C concentrations both during and after the acute illness and in Long COVID.

In two large RCTs (LOVIT-COVID NCT04401150 and REMAP-CAP NCT02735707) the possible ability of iv vitamin C to prevent Long COVID when applied during the acute illness is currently being co-investigated. In these studies, vitamin C is administered at a daily dose of 200 mg per kg bw for 4 days, and health-related quality of life is evaluated during the studies and after 6 months. The problem of the short duration of treatment also applies to these studies and probably could lead to negative or not informative results on the long-term quality of life. The relevance of treatment duration is supported by a recent nationwide cohort study that investigated iv vitamin C in sepsis and revealed a significant reduction in mortality when applied for ≥5 days (Jung et al., 2022). Therefore a more pragmatic approach is proposed in which patients receive iv vitamin C throughout their hospital stay and, after discharge, are switched to high doses of oral vitamin C in order to investigate whether this has a greater impact on Long COVID and even on structural pulmonary changes (Holford et al., 2021).

There is good study evidence that high-dose iv vitamin C can reduce fatigue, cognitive impairment and pain in conditions associated with oxidative stress. It is very plausible that these effects could also be achieved in the treatment of Long COVID patients. This should be investigated by appropriate RCTs.
